# Oscillations in MAPK cascade triggered by two distinct designs of coupled positive and negative feedback loops

**DOI:** 10.1186/1756-0500-5-287

**Published:** 2012-06-13

**Authors:** Uddipan Sarma, Indira Ghosh

**Affiliations:** 1 National Centre for Cell Science, Ganeshkhind, Pune, India; 2 School of Computational and Integrative Sciences, Jawaharlal Nehru University, New Delhi, India

## Abstract

**Background:**

Feedback loops, both positive and negative are embedded in the Mitogen Activated Protein Kinase (MAPK) cascade. In the three layer MAPK cascade, both feedback loops originate from the terminal layer and their sites of action are either of the two upstream layers. Recent studies have shown that the cascade uses coupled positive and negative feedback loops in generating oscillations. Two plausible designs of coupled positive and negative feedback loops can be elucidated from the literature; in one design the positive feedback precedes the negative feedback in the direction of signal flow and vice-versa in another. But it remains unexplored how the two designs contribute towards triggering oscillations in MAPK cascade. Thus it is also not known how amplitude, frequency, robustness or nature (analogous/digital) of the oscillations would be shaped by these two designs.

**Results:**

We built two models of MAPK cascade that exhibited oscillations as function of two underlying designs of coupled positive and negative feedback loops. Frequency, amplitude and nature (digital/analogous) of oscillations were found to be differentially determined by each design. It was observed that the positive feedback emerging from an oscillating MAPK cascade and functional in an external signal processing module can trigger oscillations in the target module, provided that the target module satisfy certain parametric requirements. The augmentation of the two models was done to incorporate the nuclear-cytoplasmic shuttling of cascade components followed by induction of a nuclear phosphatase. It revealed that the fate of oscillations in the MAPK cascade is governed by the feedback designs. Oscillations were unaffected due to nuclear compartmentalization owing to one design but were completely abolished in the other case.

**Conclusion:**

The MAPK cascade can utilize two distinct designs of coupled positive and negative feedback loops to trigger oscillations. The amplitude, frequency and robustness of the oscillations in presence or absence of nuclear compartmentalization were differentially determined by two designs of coupled positive and negative feedback loops. A positive feedback from an oscillating MAPK cascade was shown to induce oscillations in an external signal processing module, uncovering a novel regulatory aspect of MAPK signal processing.

## Background

**Figure 1:**
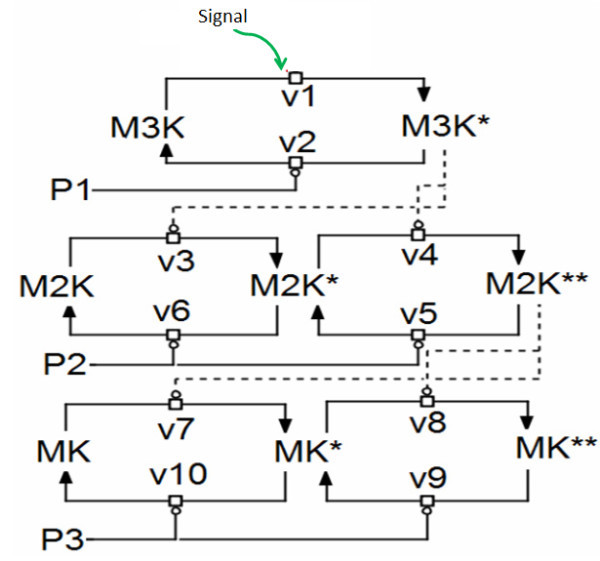
**Schematic representation of the three layer MAPK cascade.** The cascade is activated by incoming signal represented as “Signal”. The Signal phosphorylates M3K to M3K* where “*” represents phosphorylation. M3K* phosphorylates its downstream kinase M2K in two successive steps to M2K**. The doubly phosphorylated M2K** phosphorylates MK to MK** and MK** is the output of the MAPK cascade. The phosphatases P1, P2 and P3 dephosphorylate the kinases M3K, M2K and MK respectively. The rates v1-v10 in a sequential order represents the reaction fluxes. Here the solid lines with blunt heads correspond to dephosphorylation steps carried out by the phosphatases and dashed lines with blunt heads corresponds to phosphorylation of the cascade kinases by their respective upstream kinases.

Signal transduction pathways such as the Mitogen Activated Protein Kinase (MAPK) cascade responds to wide range of external stimuli to trigger growth, cell-division and proliferation [1,2]. The evolutionarily conserved structure of the three layer MAPK cascade consists of the MAPKKK (henceforth referred as M3K), MAPKK (henceforth referred as M2K) and MAPK (henceforth referred as MK) from yeast to human, which processes the incoming signal through a series of covalent modification cycles [1]. M3K is activated upon single phosphorylation whereas M2K and MK are both activated upon double phosphorylation [2-4]. Parallel to the phosphorylation by kinases, phosphatases present in the cellular volume dephosphorylates the phosphorylated kinases. Figure 1 shows the schematics of a three layer MAPK cascade where each layer of the cascade is dephosphorylated by a specific phosphatase. Phosphorylated M3K (M3K*) is dephosphorylated by a phosphatase P1, phosphorylated forms of M2K (singly phosphorylated M2K* and doubly phosphorylated M2K**) are dephosphorylated by P2 and phosphorylated forms of MK (singly phosphorylated MK* and doubly phosphorylated MK**) are dephosphorylated by a phosphatase P3. Various feedback loops, both positive and negative in nature are abundant in the biological signal processing pathways. In a three layer MAPK cascade both positive and negative loops are found to be operational [5]. 

Coordinated actions of coupled positive and negative feedback loops have been reported earlier for biochemical systems with different architectural designs. In cyclin-dependent kinase 1 (CDK1) pathway, coupled positive and negative feedback loops leads to robust oscillations where time periods of oscillations can be changed without compromising the amplitude of oscillations [6]. In another study, it was found that during calcium spike regulation, positive feedback loops constituting IP3R and RYR and a negative feedback loop constituting SERCA ATPases triggers and regulates the Ca2^++^ oscillations [7]. Similarly the cell cycle oscillations are essentially built from coupled positive and negative feedback loops between Cdc2 and APC system that gives reliable cell cycle oscillations [8]. The p53 pathway which is another oscillatory pathway is also densely wired in positive and negative feedback loops [9]. A recent experimental study shows that a negative feedback from the phosphorylated ERK (MK**) to its upstream activator SOS and a coupled positive feedback from MK** to M3K* (by phosphorylation mediated dissociation of M3K* complex from its inhibitor RKIP) results in robust system-level oscillations [5], suggesting for the first time that the MAPK pathway can employ coupled positive and negative feedback loops for generating its oscillations.

In the three-layer MAPK cascade, both positive and negative feedback loops emerges from the fully phosphorylated MK (MK**). Feedback loops from MK** act on its upstream M2K and M3K layers and alter their phosphorylation according to the nature of the feedback loop. A list of feedback loops reported to be operative between MK & M2K or MK & M3K are listed in Table 1. It implies from the Table 1, that two distinct designs of coupled positive and negative feedbacks can potentially exist in the three layer MAPK cascade. One design comprises a negative feedback from MK** to M3K phosphorylation coupled to a positive feedback from MK** to M2K phosphorylation, which we named as PN-I design. The other design shows a positive feedback from MK** to M3K phosphorylation coupled to negative feedback from MK** to M2K phosphorylation which is represented as PN-II design. Although it is observed that coupling of both positive and negative feedback loops can trigger oscillations in the MAPK cascade [5], potential of both the designs for generating oscillations in the MAPK cascade remains to be elucidated. 

**Table 1:** Schematics of positive and negative feedback loops operational in MAPK cascade

**Feedback interaction of kinases**	**Feedback types**	**References**
Sub module 1	Positive	25, 46
Sub module 2	Negative	41, 47
Sub module 3	Positive	16
Sub module 4	Negative	24

Also during long duration signaling, MK and its phosphorylated forms (MK* and MK**), traverses between cytoplasm and nucleus [10-12]. Inside the nucleus, MK** induces expression of its phosphatase (MKP-1) that subsequently carries out MK** dephosphorylation in the nucleus itself [4,10]. It is not known how nuclear-cytoplasmic shuttling of the terminal layer kinase of MAPK cascade and the subsequent transcriptional induction of phosphatase such as MKP-1 would affect the oscillations triggered by PN-I and PN-II.

Here we built two oscillating models of MAPK cascade where oscillations in one model were triggered by PN-I and the oscillations in the other model were triggered by PN-II. We found that in both the cases, the amplitude, frequency and nature (digital/analogous) of oscillations were uniquely shaped by the coupled positive and negative feedback loops embedded in the cascade. Our simulations show that the MAPK cascade embedded in PN-II exhibited remarkable robustness in generating oscillations with identical frequency and amplitude while subjected to a wide range of input stimuli, whereas, the cascade embedded in PN-I was less robust in maintaining its frequency and amplitude when subjected to input signal of different strengths. We also found that a positive feedback emerging from an oscillating MAPK cascade and functional in a different pathway or signaling module could lead to both signal amplification and oscillations in the external module. Further we investigated the fate of oscillations of the MAPK cascade considering the nuclear and cytoplasmic shuttling. Our analysis revealed that the oscillations of the MAPK cascade embedded in PN-I were not affected by such shuttling of the cascade components and induction of its nuclear phosphatase, whereas oscillations triggered by PN-II were completely abolished when induction of nuclear phosphatase was considered. Sensitivity analysis for small perturbations in parameters of the oscillating models were carried out which showed that the organization of the feedbacks (PN-I or PN-II) also distinctly determines the most sensitive kinetic parameters in the oscillating systems. Biological significance of our findings is discussed.

## Methods

### I. Model building

Information in the signaling cascades such as MAPK cascade propagates as a result of phosphorylation-dephosphorylation of the kinases in the cascade. Upon external stimulation, M3K is phosphorylated once and the M3K* acts as an enzyme in phosphorylating its downstream kinase M2K. M3K* doubly phosphorylates M2K in two single phosphorylation steps [3]. Similarly, the doubly phosphorylated M2K (M2K**) phosphorylates MK to MK** in two steps. MK** is the output of the MAPK cascade. Every layer of the cascade has its individual phosphatase [3], which carries out the dephosphorylation process concurrent to the phosphorylation process. Let’s assume an external signal ‘Signal’ triggers the phosphorylation of M3K and a cellular phosphatase P1 dephosphorylates the phosphorylated M3K* back to its unphosphorylated form. The biochemical reaction for phosphorylation process is given as

Signal+M3K⇔[Signal.M3K]→Signal+M3K*

And the biochemical reaction for dephosphorylation process is given as

P1+M3K*⇔[P1.M3K*]→P1+M3K

In the phosphorylation reaction, the ‘Signal’ could be an upstream kinase [13] or other activators that triggers M3K phosphorylation [3,10]. The phosphorylation-dephosphorylation cycles follow in the M2K and MK layers and the cascade delivers its final output MK**.

Under the steady state of production and degradation of [Signal.M3K] and [P1.M3K*], flux equations of M3K phosphorylation and dephosphorylation (v1 and v2 in Figure 1) can be given as [14,15]

(1) $$v1=\frac{\frac{k_{1}.\text{Signal}.M3K}{K1}}{\left(1+\frac{M3K}{K1}\right)}=\frac{\frac{\text{Sig}.M3K}{K1}}{\left(1+\frac{M3K}{K1}\right)}$$

(2) $$v2=\frac{\frac{k_{2}.P1.M3K*}{K2}}{\left(1+\frac{M3K*}{K2}\right)}$$

Where $Sig=k_{1}.Signal$, k_1_ and k_2_ are the catalytic rates associated with the phosphorylation and dephosphorylation processes respectively. K1 and K2 are the Km values of the reactions. Phosphorylation-dephosphorylation reactions for the M2K and MK layer takes place in two steps and the equations could be derived accordingly assuming steady state conditions [14,15].

In MAPK cascade, both positive and negative feedback loops emerge from MK** and are functional in either of the two upstream layers, M2K and M3K (Table 1). Thus the flux equations will be modified in presence of these feedback loops.

Phosphorylation of M3K as shown in equation (1) would be modified in presence of a negative feedback loop as,

(3) $$v1_{\mathrm{neg}}=\frac{\frac{\text{Sig}.M3K}{K1}}{\left(1+\frac{M3K}{K1}).(1+{\left(\frac{MK**}{KI}\right)}^{n1}\right)}$$

In equation (3), ‘KI’ captures the strength of negative feedback of MK** on M3K phosphorylation. The negative feedback is assumed as a hyperbolic modifier, which is non competitive in nature and ‘n1’ is the associated Hill coefficient [14]. The subscript ‘neg’ associated with v1 in equation (3) represents phosphorylation in presence of negative feedback.

In presence of the positive feedback loop, the flux of M3K phosphorylation is modified as

(4) $$v1_{\mathrm{pos}}=\frac{\left(\text{Sig}.\frac{M3K}{K1}).(1+{\left(\frac{A.MK**}{Ka}\right)}^{n1}\right)}{\left(1+\frac{M3K}{K1}).(1+{\left(\frac{MK**}{Ka}\right)}^{n1}\right)}$$

In equation (4), A and Ka are the kinetic constants associated with the positive feedback from MK** to the M3K layer phosphorylation [16]. The subscript ‘pos’ associated with v1 in equation (4) represents phosphorylation in presence of positive feedback. In equation (4) the exponent n1 is the Hill coefficient which indicates that the positive feedback is a hyperbolic modifier of the M3K phosphorylation [16]. The positive feedback was assumed as a hyperbolic modifier in all the model equations involving the positive feedback.

A set of coupled ordinary differential equations capture the signal flow in the MAPK cascade which are given as

$$\begin{array}{l} \frac{d[M3K*]}{dt}=v1-v2\\ \frac{d[M2K*]}{dt}=v3-v4+v5-v6\\ \frac{d[M2K**]}{dt}=v4-v5\\ \frac{d[MK*]}{dt}=v7-v8+v9-v10\\ \frac{d[MK**]}{dt}=v8-v9 \end{array}$$

The vi, i = 1-10 are the flux equations as given in Table 2 and also shown schematically in Figure 1. The amount of M3K, M2K and MK at any point of time can be calculated from the following mass conservation equations.

$$\begin{array}{l} \;{\left[M3K\right]}_{\mathrm{Total}}=[M3K]+[M3K*]\\ {\left[M2K\right]}_{\mathrm{Total}}=[M2K]+[M2K*]+[M2K**]\\ {\left[MK\right]}_{\mathrm{Total}}=[MK]+[MK*]+[MK**] \end{array}$$

**Table 2:** Flux of signal flow in cytoplasmic MAPK cascades ‘S1’ and ‘S2’

**Model ** **reactions**	**Flux equations in model S1**	**Flux equations in model S2**
1] M3K→M3K*	$v1_{\mathrm{neg}}=\frac{\frac{Sig.M3K}{K1}}{\left(1+\frac{M3k}{K1}).(1+{\left(\frac{{MK}^{**}}{KI}\right)}^{n1}\right)}$	$v1_{\mathrm{pos}}=\frac{(Sig.\frac{M3K}{K1}).(1+{\left(\frac{A.{MK}^{**}}{Ka}\right)}^{n}}{(1+\frac{M3K}{K1}).(1+{\left(\frac{{MK}^{**}}{Ka}\right)}^{n1}}$
2] M3K*→ M3K	$v2=\frac{k2.P1\frac{{M3K}^{*}}{K2}}{1+\frac{{M3K}^{*}}{K2}}$	$v2=\frac{k2.P1.\frac{{M3K}^{*}}{K2}}{1+\frac{{M3K}^{*}}{K2}}$
3] M2K→M2K*	$v3_{\mathrm{pos}}=\frac{\left(k3.{M3K}^{*}.\frac{M2K}{K3}).(1+{\left(\frac{A.{MK}^{**}}{Ka}\right)}^{n3}\right)}{\left(1+\frac{{M3K}^{*}}{K4}+\frac{M2K}{K3}).(1+{\left(\frac{{MK}^{**}}{Ka}\right)}^{n3}\right)}$	$v3_{\mathrm{neg}}=\frac{\left(k3.{M3K}^{*}.\frac{M2K}{K3}\right)}{\left(1+\frac{{M2K}^{*}}{K4}+\frac{M2K}{K3}).(1+{\left(\frac{{MK}^{**}}{KI}\right)}^{n3}\right)}$
4] M2K*→M2K**	$v4_{\mathrm{pos}}=\frac{\left(k4.{M3K}^{*}.\frac{M2K*}{K4}).(1+{\left(\frac{A.{MK}^{**}}{Ka}\right)}^{n4}\right)}{\left(1+\frac{M2K*}{K4}+\frac{M2K}{K3}).(1+{\left(\frac{{MK}^{**}}{Ka}\right)}^{n4}\right)}$	$v4_{\mathrm{neg}}=\frac{\left(k4.{M3K}^{*}.\frac{M2K*}{K4}\right)}{\left(1+\frac{{M2K}^{*}}{K4}+\frac{M2K}{K3}).(1+{\left(\frac{{MK}^{**}}{KI}\right)}^{n4}\right)}$
5] M2K**→ M2K*	$v5=\frac{\frac{k5.P2.{M2K}^{**}}{K5}}{1+\frac{M2K**}{K5}+\frac{M2K*}{K6}}$	$v5=\frac{\frac{k5.P2.{M2K}^{**}}{K5}}{1+\frac{{M2K}^{**}}{K5}+\frac{{M2K}^{*}}{K6}}$
6] M2K*→ M2K	$v6=\frac{\frac{k6.P2.{M2K}^{*}}{K6}}{1+\frac{M2K*}{K6}+\frac{{M2K}^{**}}{K5}}$	$v6=\frac{\frac{k6.P2.M2K*}{K6}}{1+\frac{{M2K}^{*}}{K6}+\frac{{M2K}^{**}}{K5}}$
7] MK→ MK*	$v7=\frac{\frac{k7.M2K**.MK}{K7}}{1+\frac{MK}{K7}+\frac{{MK}^{*}}{K8}}$	$v7=\frac{\frac{k7.{M2K}^{**}.MK}{K7}}{1+\frac{MK}{K7}+\frac{{MK}^{*}}{K8}}$
8] MK*→ MK**	$v8=\frac{\frac{k8.M2K**.{MK}^{*}}{K8}}{1+\frac{MK*}{K8}+\frac{MK}{K7}}$	$v8=\frac{\frac{k8.{M2K}^{**}.{MK}^{*}}{K8}}{1+\frac{{MK}^{*}}{K8}+\frac{MK}{K7}}$
9] MK** → MK*	$v9=\frac{\frac{k9.P3.{M3K}^{**}}{K9}}{1+\frac{{MK}^{**}}{K9}+\frac{{MK}^{*}}{K10}}$	$v9=\frac{\frac{k9.P3.{MK}^{**}}{K9}}{1+\frac{{MK}^{**}}{K9}+\frac{{MK}^{*}}{K10}}$
10] MK* → MK	$v10=\frac{\frac{k10.P3.{MK}^{*}}{K10}}{1+\frac{{MK}^{**}}{K9}+\frac{{MK}^{*}}{K10}}$	$v10=\frac{\frac{k10.P3.MK*}{K10}}{1+\frac{{MK}^{**}}{K9}+\frac{{MK}^{*}}{K10}}$

The fluxes of signal flow and the values of kinetic parameters used for simulation of S1, S2, S1n and S2n are shown in Table 2. In this table, Ki, i = 1–10 are the Km values of the reactions and ki, i = 2–10 are the kcat values of the reactions. The numerical value of ‘i’ corresponding to Ki and ki represents the reaction number, KI are the kinetic parameters associated with negative feedback. Ka and A are the kinetic constants associated with the positive feedback. The hill coefficient used in the equations 1, 3 and 4 are shown as n1, n3 and n4 respectively.

As the total concentration of a kinase is known, M3K, M2K and MK can be calculated from the above mass conservation equations and the differential equations.

### Models S1 and S2

**Figure 2:**
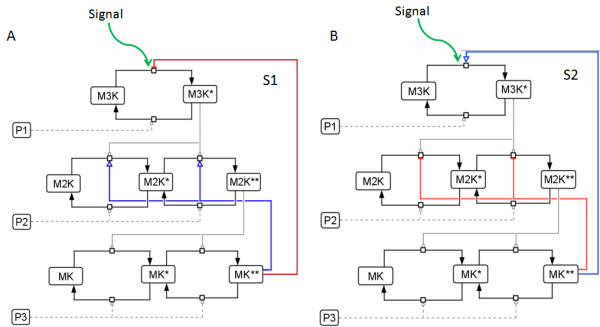
**Schematic representation of the cascades S1 and S2.** The MAPK cascades embedded in two differential designs of positive and negative feedback loops are shown here. **(A)** An external signal ‘Signal’ activates the cascade S1 by triggering the phosphorylation of M3K. The cascade is embedded in the feedback design PN-I. Negative feedback from MK** to M3K layer is shown with a red bar with blunt head and positive feedback from MK** to M2K layer is shown with blue arrows. **(B)** The external signal ‘Signal’ triggers M3K phosphorylation and the cascade S2 embedded in the feedback design PN-II is activated. Positive feedback from MK** to the M3K layer and negative feedback from MK** to the M2K layer are shown as blue arrow and red bars, respectively.

Based on different types of positive and negative feedback loops reported (shown in Table 1), two distinct designs of coupled positive and negative feedback loops emerges. Figure 2 shows the two designs of coupled positive and negative feedback loops functional in a MAPK cascade, namely S1 and S2. S1 comprises negative feedback from MK** to M3K layer coupled to positive feedback from MK** to M2K layer. In S2, negative feedback from MK** to M2K layer is coupled to positive feedback from MK** to M3K layer. The flux equations of models S1 and S2 are given in Table 2. All the flux equations corresponding to dephosphorylation are identical to each other in both S1 and S2. Also the flux equations of phosphorylation corresponding to MK layer are identical in both S1 and S2. Both S1 and S2 were simulated to understand the significance of PN-I and PN-II designs in generating oscillations in the MAPK cascade. We studied the characteristic frequency, amplitude and robustness of the oscillations triggered by designs, PN-I and PN-II.

### Modification of the models S1 and S2 to incorporate nuclear-cytoplasmic shuttling

Nuclear-cytoplasmic shuttling of the MK layer components (MK, MK* and MK**) of the MAPK cascade takes place [10-12] where MK** triggers various transcription factors in the nucleus, aiming to activate target genes [10,17]. We updated the models S1 and S2 to S1n and S2n respectively, to incorporate the nuclear-cytoplasmic translocation of the MK layer components of the cascade.

In both the modified models, MK** translocate to the nucleus and induces its own phosphatase MKP-1 (named P3-n in the models). The biochemical reactions and flux equations corresponding to MK layer’s nuclear-cytoplasmic shuttling and the transcriptional induction of P3-n were adopted from a recent study [10], which is given in Table 3. The models S1n and S2n comprise of 22 flux equations where the first 10 equations in S1n and S1 are identical to each other which are given in Table 2. Similarly the first 10 flux equations of model S2n are identical to that of model S2 (Table 2). The additional equations shown in Table 3 incorporates the nuclear cytoplasmic shuttling of the MK layer components MK, MK* and MK**. These also include the equations that capture the induction of mRNA of P3-n from the target gene triggered by MK** in the nucleus and the subsequent biochemical steps that leads to P3-n production. The transcriptionally induced phosphatase P3-n dephosphorylates MK* and MK** in the nucleus. The differential equations corresponding to the modified section of the model can be found in the Additional file 1: model files S1n and S2n. The mass conservation equations are identical for S1, S2, S1n and S2n. 

**Table 3:** After addition of the nuclear components, ‘S1’ and ‘S2’ were renamed as ‘S1n’ and ‘S2n’ respectively

**Reaction number in models S1n and S2n**	**Reaction**	**Flux equation**	**Parameter value**
11]	MK** ↔ MK**-n	k11_f_.MK** - k11_b_.MK**-n	k11_f_ = 10.34 sec^-1^
			k11_b_ = 2.86 sec^-1^
12]	P3-n gene → PreP3mRNA	$\frac{V12.{\left({MK}^{**}-n\right)}^{n12}}{{\left(K12\right)}^{n12}+{\left({MK}^{**}-n\right)}^{n12}}$	V12 = 29.24 nmol/sec
			K12 = 169 nmol/ml
			n12 = 3.97
13]	PreP3mRNA→P3mRNA	k13. PreP3mRNA	k13 = 0.022 sec^-1^
14]	P3mRNA → ɸ	k14.P3mRNA	k14 = 0.0078 sec^-1^
15]	P3mRNA → P3-c	k15.P3mRNA	k15 = 0.0012 sec^-1^
16]	P3-c ↔ P3-n	k16_f_.P3-c - k16_b_.P3-n	k16_f_ = 22.56 sec^-1^
			k16_b_ = 15.4 sec^-1^
17]	P3-c → ɸ	k17.P3-c	k17 = 0.00025 sec^-1^
18]	P3-n → ɸ	k18.P3-n	k18 = 0.00025 sec^-1^
19]	MK ↔ MK-n	k19_f_.MK - k19_b_.MK-n	k19_f_ = 10.34 sec^-1^
			k19_b_ = 2.86 sec^-1^
20]	MK* ↔ MK*-n	k20_f_.MK* - k20_b_.MK*-n	k20_f_ = 10.34 sec^-1^
			k20_b_ = 2.86 sec^-1^
21]	MK**-n → MK*-n	$\frac{k21.(P3-n).\frac{\left({MK}^{**}-n\right)}{K21}}{1+\frac{{MK}^{**}-n}{K21}+\frac{{MK}^{*}-n}{K22}}$	k21 = 0.68 sec^-1^
			K21 = 10300 nmol/ml
			K22 = 87 nmol/ml
22]	MK*-n → MK-n	$\frac{k22.(P3-n).\frac{\left({MK}^{*}-n\right)}{K22}}{1+\frac{{MK}^{*}-n}{K22}+\frac{{MK}^{**}-n}{K21}}$	k22 = 0.31 sec^-1^
			K21 = 10300 nmol/ml
			K22 = 87 nmol/ml

Table shows the reactions and flux equations in both S1n and S2n are shown in Table 3. Reactions 1–10 in S1n and S2n were identical to that given in Table 2 for S1 and S2 respectively. Reactions and flux equations shown in this table are common for both S1n and S2n. Reactions 21 and 22 were built from the original mass action reactions ( [10], by assuming steady state condition of the enzyme-substrate complexes, which were used to calculate Km value for reactions 21 and 22. In the reactions, ki_f_ and ki_b_ where i = 11, 16, 19, 20 are the forward and backward reaction rates of the respective reactions. The parameter values ki, where i = 13, 14, 15, 17, 18, 21, 22 are the catalytic rates of the respective reactions. Ki, i = 12, 21, 22 are the Km values of the respective reactions. V12 is the maximum velocity of the reaction 12.

### II. Model assumptions

In substantiation with the previous studies [14,15], it was assumed that a steady state in the enzyme-substrate complexes is achieved during the signal propagation, for all the reactions in both S1 and S2. For the sake of simplicity we assumed that no degradation and production of the cascade components (kinases and phosphatases) of S1 and S2 takes place during the course of signal propagation and hence their concentrations remain constant. However, following experimental guidelines [10], the models S1n and S2n were built with certain degradation and phosphatase (P3-n) production steps, as shown in Table 3. In models S1 and S2 we also assumed that each layer of the cascade is phosphorylated by one phosphatase specific to each layer [3,14], except, in the models S1n and S2n, where dephosphorylation of the third layer MK was carried out by two phosphatases, P3 and transcriptionally induced P3-n. The model presented here represents a three layer MAPK cascade that is evolutionarily conserved from yeast to mammal [1]. Although differences in the rewiring of the kinases-phosphatases interaction are observed in some eukaryotic systems [13-15,18], the kinases-phosphatases interaction shown here represents the most generalized structure of the cascade known till now [1-3]. The simplifications also included ignoring various intra modular crosstalks which involve MAPK cascade and other signaling modules [19]. While building the flux equations for positive and negative feedback loops we assumed that both the feedback types are hyperbolic modifiers, which is in corroboration with earlier studies [14,16].

### III. Model parameters and concentrations

The kcat and Km values for S1, S2, S1n and S2n were chosen in biochemically observed ranges [3,14-16,18]. Additional file 2: Table S1 describes the reactions capturing signal flow in the three layer MAPK cascade and their kinetic parameter values, which are common in all the four models S1, S2, S1n and S2n. Additional file 2: Table S2 describes the concentration of kinases and phosphatases used in S1, S2, S1n and S2n. Table 3 shows the additional reaction parameters corresponding to the modified fraction of the models S1n and S2n. Parameters for the additional reactions in the model S1n and S2n were adopted from a recent study [10].

### IV. Sensitivity analysis for small perturbations in the model parameters

Sensitivity studies reveal the relative importance of kinetic parameters associated with the model. We performed sensitivity analysis of all the four models by applying small perturbations to the kinetic parameters of the models and measuring the sensitivity of MK** in each of the model to such perturbations.

Mathematically, the sensitivity coefficients are the first order derivatives of model outputs with respect to the model parameters; $S_{\mathrm{ij}}=\frac{\partial O_{i}}{\partial p_{j}}$, where O_i_ is the i^th^ model output and p_j_ is the j^th^ model parameter [20,21]. S_ij_ is the sensitivity coefficient which yields sensitivity of O_i_ with respect to the perturbation in parameter p_j_. We have calculated the sensitivity coefficient S_ij_ using the software SBML-SAT that implements the centered difference assumption for calculating S_ij_[21]. When a parameter p_j_ is subjected to a small perturbation (∆p_j_) in its reference value (reference value is the unperturbed value), the sensitivity coefficient S_ij_ is calculated as

$$S_{\mathrm{ij}}=\frac{O_{i}(p_{j}+\Delta p_{j})-O_{i}(p_{j}-\Delta p_{j})}{2\Delta p_{j}}$$

Upon normalization, the sensitivity coefficient S_ij_ is given as: $S_{\mathrm{ij}}{}^{\mathrm{normalized}}=\frac{\frac{O_{i}(p_{j}+\Delta p_{j})-O_{i}(p_{j}-\Delta p_{j})}{O_{i}}}{\frac{2\Delta p_{j}}{p_{j}}}$

In the above equation, we calculated S_ij_ with ∆p_j_ = 0.001*p_j_ for any perturbed parameter p_j_. The variation of ∆p_j_ in the range of 0.0001*p_j_-0.1*p_j_ didn’t alter S_ij_. The perturbations were applied locally, which means parameters were perturbed one at a time and S_ij_ for each of the parameter’s perturbation on the output MK** of the models was calculated.

### V. Software used and model simulations

For performing the simulations SBML models were initially constructed using Complex pathway simulator (COpasi) [22]. The time course simulations were carried out in COpasi. Sensitivity analysis was performed using SBML_SAT, a MATLAB toolbox for sensitivity analysis [21]. Bifurcation analysis to inspect oscillation in S2n was carried out using Bifurcation Discovery tool [23]. The model files are given as additional files.

## Results

We constructed two models S1 and S2 of the MAPK cascade, one embedded in PN-I and the other embedded in PN-II respectively, such that oscillations in both the models were triggered by coupled positive and negative feedback loops. We investigated the fate of MAPK oscillations in S1 and S2, when signal strength was varied in wide ranges. Our simulations also revealed that MAPK cascade can utilize its positive feedback to trigger oscillations in an external signal processing module. Next we examined the fate of oscillations triggered by PN-I and PN-II when nuclear–cytoplasmic shuttling of the components of terminal layer MK of the MAPK cascade takes place followed by the induction of a nuclear phosphatase by MK**. Results show that oscillations triggered by PN-II exists only in the cytoplasm and induction of the P3-n completely abolished the oscillations, whereas oscillations triggered by PN-I are not affected by the nuclear translocation of MK layer and subsequent induction of nuclear phosphatase. Various in-silico knock out studies were carried out to elucidate the importance of cytoplasmic and nuclear phosphatases in both S1 and S2. Also, when the parameters of S1, S1n, S2 and S2n were subjected to small perturbations, we found that PN-I and PN-II differentially regulates the cascades’ output sensitivity to these perturbations.

### Oscillations in models S1 and S2

Previous studies show that negative feedback from MK** to M3K layer (Sub module 2, Table 1) [14], or negative feedbacks from MK** to M2K layer (Sub module 4, Table 1) [24], triggers sustained oscillations in the MAPK cascade. Positive feedback from MK** to M3K phosphorylation (Sub module 1 Table 1) results in all-or-none behavior in production of MK** [4,25,26]. Positive feedback from MK** to M2K phosphorylation step (Sub module 3, Table 1) was found to facilitate propagation of long range phosphorylation waves of MK** in the developing neurons [16]. Earlier computational investigations revealed that a negative feedback from MK** to M3K layer is a prerequisite in triggering MAPK oscillations [14], but later it was found that for certain parameter combinations, the three layer MAPK cascade can trigger its oscillations in absence of the explicit negative feedback loop from MK** to M3K [27].

But a recent experiment exposed that MAPK oscillations are triggered by coupled positive and negative feedback loops [5]. This experimental finding necessitated an investigation on the significance of differential designs of coupled positive and negative feedback loops that can plausibly trigger oscillations in the cascade and the characteristics of oscillations triggered by each of the design. The MAPK cascades embedded in the two designs of coupled positive and negative feedback loops, PN-I and PN-II are shown in Figure 2A and 2B.

**Figure 3:**
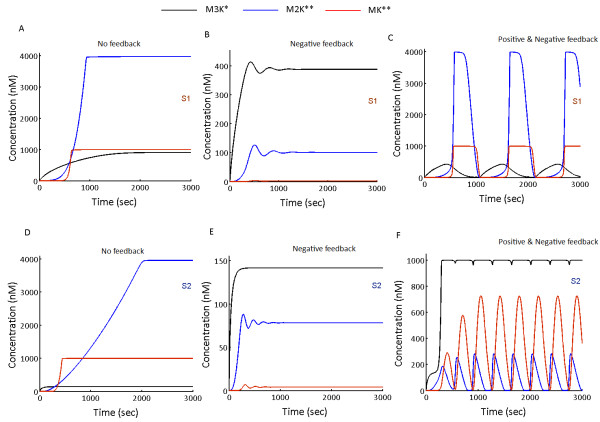
**Dynamics of MAPK cascade embedded in PN-I and PN-II.** Both models S1 and S2 were simulated without any feedbacks, with negative feedback alone and with coupled positive and negative feedbacks. Without any feedback loop, both S1 and S2 shows full amplitude phosphorylation of MK**, M2K** and M3K* (**A** and **D**). The negative feedback from MK** to M3K phosphorylation in S1 [**B**] and from MK** to M2K phosphorylation in S2 (**E**) resulted in inhibition of MK** amplitude. The coupled feedback design PN-I in S1 (**C**) and PN-II in S2 triggered sustained oscillations (**C** &**F**).

Upon simulation of models S1 and S2 without any feedback loops, maximum amplitude phosphorylation of the output (MK**) was attained (Figure 3A and 3D). When both the models were simulated in presence of only negative feedback loops, MK** amplitude was inhibited (Figure 3B and Figure 3E). This occurs as a result of negative feedback mediated suppression of M3K layer phosphorylation in S1 and M2K layer phosphorylation in S2 respectively. We show that the employed negative feedbacks lead to inhibition of MK** amplitude in both S1 and S2, to demonstrate that the models considered in our studies do not oscillate only in the presence of the negative feedback loop like the earlier reports [14,24]. Next when positive feedbacks were introduced in the models, both S1 and S2 exhibited sustained oscillations (Figure 3C and 3F), demonstrating that the MAPK cascades considered for our study oscillate only in the presence of coupled positive and negative feedback loops. 

### Oscillations in S1

Introduction of the positive feedback loop from MK** to M2K layer in the cascade with negative feedback from MK** to M3K layer first resulted in enhancement of the amplitude of M2K** followed by enhancement in MK** amplitude. Since both positive and negative feedbacks emerges from MK**, enhanced MK** amplitude results in stronger inhibition in the M3K* layer and stronger activation in the M2K layer. However as M3K lies upstream to M2K, decrease in M3K* concentration beyond a certain threshold results in attenuation of M2K layer phosphorylation, even in the presence of the positive feedback loop. With inhibition of M2K** amplitude, phosphorylation of MK layer gets inhibited. With decrease in MK layer phosphorylation, attenuation of the strengths of both positive and negative feedback loops follow. As MK** amplitude reaches its lowest amplitude, one cycle of oscillation is completed (Figure 3C). As the input signal is available for M3K phosphorylation, M3K* starts building up in absence of the negative feedback and the next cycle of oscillation is triggered. The process continues until the external signal is available to phosphorylate M3K. Coupling of inhibitory and activating effects of the PN-I, triggered oscillations (Figure 3C) in all the three kinases of the MAPK cascade S1.

### Oscillations in S2

Oscillations in S2 emerged due to positive feedback mediated enhancement of M3K* amplitude coupled to the negative feedback mediated inhibition of M2K**. Upon stimulation of the cascade by external signal, positive feedback from MK** to M3K enhanced the M3K* amplitude. This subsequently enhances M2K layer phosphorylation (in presence of the negative feedback from MK** to the M2K layer), ultimately resulting in amplification of MK** amplitude. Amplified MK** subsequently enhances the strengths of both positive and negative feedback loops. When MK** reaches its maximum phosphorylation amplitude (Figure 3F), negative feedback mediated inhibition of M2K layer phosphorylation surmounts the positive feedback mediated enhancement of M2K layer phosphorylation by M3K*. With progressive attenuation of M2K** amplitude, MK layer phosphorylation gets inhibited until it reaches its lowest phosphorylation amplitude (Figure 3F). The whole process completes one cycle of oscillation. The next cycle of oscillation starts when the external signal triggers phosphorylation of M3K in absence of the negative feedback from MK**. It could be noted that the negative feedback in S2 inhibits MK** production in two ways, firstly by directly inhibiting the M2K** amplitude and secondly by indirectly inhibiting the M2K** by attenuating the strength of positive feedback loop from MK** to the M3K layer. The study additionally uncovered that positive feedback not only enhanced M3K* amplitude but it also triggered oscillations in M3K* (Figure 3F).

### Nature of oscillations in S1 and S2

In S1, where the incoming signal encounters the negative feedback first and then the positive feedback, output oscillations (MK**) are digital in nature (Figure 3C). In S2, the signal encounters positive feedback first followed by its encounter with the negative feedback, which resulted in sinusoidal oscillations (Figure 3F). In the MAPK cascade, it is known that positive feedback stabilizes [25,28] and negative feedback destabilizes [14,24] the output (MK**) amplitude. Here we showed that the interplay between such stabilizing and destabilizing effect differentially determines the nature of oscillations which ultimately depends on the designs of coupled feedback loops. The digital oscillations in S1 exhibited sharp switch like characteristics of a positive feedback [29] in the rise and fall of the phosphorylation waves (Figure 3C) and the analogous oscillations in S2 exhibited characteristics of a negative feedback mediated oscillations observed earlier [11]. The study suggests that output characteristics of an oscillating MAPK cascade is based on the feedback type encountered by the incoming signal at the M2K layer.

Next we examined how oscillations in the MAPK cascade embedded in PN-I and PN-II are affected when both S1 and S2 are activated by input signal of different strengths.

### Oscillations in S1 and S2 subjected to a wide range of input stimuli

Signal strength varies widely in the in-vivo conditions. The strength of the incoming signal is governed by the concentration of the signal as well as the proximity of the signal source to the target receptor that activates a signaling pathway [3,5,30,31]. However biological systems are built to maintain their output characteristics in the face of perturbations [32]. Thus we examined the relative robustness of S1 and S2 in triggering their characteristic oscillations when both the systems were subjected to a spectrum of input signals.

### I. Model S1

**Figure 4:**
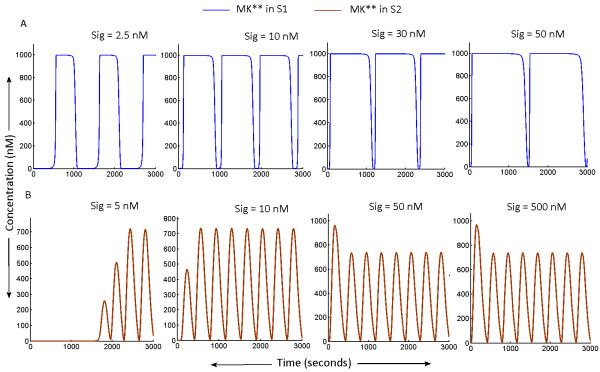
**Sustained oscillations in S1 and S2 as function of signal dose.** Strength of the signal activating the MAPK cascade ‘Sig’ was varied and the range of ‘Sig’ in which both S1 and S2 would exhibit oscillations was studied. **(A)** After a certain threshold value of ‘Sig’, increase in ‘Sig’ resulted in decrease in the frequency of MK** oscillations in S1, whereas the maximum amplitude of oscillations remained unaltered. When the range of ‘Sig’ triggering such oscillations was crossed beyond 50nM, the oscillations ceased to exist (data not shown). **(B)** In S2, beyond a threshold value of Sig (~ 5 Nm) required to trigger oscillations, almost any value of ‘Sig’ triggered oscillations with conserved frequency and amplitude.

Figure 4A shows the oscillation characteristics of S1 subjected to a range of input signals (varied between 2.5 nM- 50 nM; 2.5 nM is the minimum signal strength required for triggering the oscillations). At a low signal strength, (shown as ‘Sig’ in Figure 4A) MK** oscillations with maximum amplitude were achieved. With increase in signal strength, the effect of negative feedback mediated suppression of M3K phosphorylation was diluted and beyond a certain strength of the input signal (Sig > 50 nM), the negative feedback can no longer suppress M2K layer phosphorylation by inhibiting M3K phosphorylation. Thus beyond a certain strength of input signal (Sig > 50 nM), coupled effect of the strong input signal and the positive feedback from MK** to M2K layer resulted in a steady non-oscillatory phosphorylation of M2K** and MK** (data not shown). However if the signal was applied in the range provided above, sustained oscillations could be achieved in the cascade’s output phosphorylation (Figure 4A). With increase in signal strength (in the range 2.5-50 nM), oscillation amplitudes were conserved, but the frequency of oscillations decreased with increasing strengths. Thus a MAPK cascade embedded in PN-I can exhibit conserved amplitude oscillations whose frequencies would be decided by the strengths of the incoming signal.

### II. Model S2

The model S2 was subjected to signals of variable strengths. Beyond a certain threshold (Sig ~ 5 nM) that triggered oscillations in the cascade, oscillations were observed for signals of any given strength (we tested the Sig range in 5 nM – 5000000 nM) of incoming signal. Figure 4B shows MK** oscillations in S2 for the signal strength 5-500 nM. S2 also exhibited sustained oscillations with equal frequency and amplitude for all the strengths of applied signal above the threshold strength. The causality behind emergence of such robust oscillations could emerge from the design of the coupled feedback loops. In S2, positive feedback enhances M3K* amplitude and thus for a relatively smaller signal dose M3K* reaches its maximum amplitude and saturates. Hence when the signal strength is increased further, no additional changes will be observed in the M3K layer. Since the strengths of the feedback loops becomes unresponsive to the further increases in signal strength, MK** oscillations with robustly conserved amplitude and frequency could be generated for a very wide range of input signals.

As shown earlier for the system S2, positive feedback led to oscillations in the M3K* amplitude in addition to the amplification in its phosphorylation (Figure 3F). We next investigated whether the positive feedback component of S2 (and also S1) is capable of transferring oscillations to external signal transduction modules in general.

### Positive feedback transfers oscillations from an oscillating MAPK cascade to other signaling modules

Results shown in Figure 3F opens up a possibility that positive feedback loop emerging from an oscillating MAPK cascade could trigger oscillations in its place of action in addition to the signal amplification in the target site. Experimentally such positive feedback loop is observed from the output MK** (from p38MAPK cascade) to the p53 phosphorylation step [9]. Similarly positive feedback from the output MK** (from ERK cascade) leads to modification of Lck kinase as observed in the T lymphocytes [33]. We investigated how the positive feedback from oscillating MAPK cascades such as S1 or S2 would affect the phosphorylation in an external signal transduction module, by building a hypothetical phosphorylation-dephosphorylation cycle with a kinase X and its phosphorylated form X-P. The model used for simulation of the positive feedback from S2 to X is provided as an additional SBML model file.

We built a model where MK** of system S2 provides a positive feedback to the phosphorylation of a kinase X (X is a hypothetical kinase phosphorylated by a different signal). Kinase X was assumed to be activated by phosphorylation like most of the kinases in the signaling networks. Also we assumed that a cellular phosphatase dephosphorylates phosphorylated X (X-P) back to its unphosphorylated form. This simple one step covalent modification cycle represents the most fundamental module of signal transduction [34] and is a building block of nearly all the signal processing modules [35,36].

**Figure 5:**
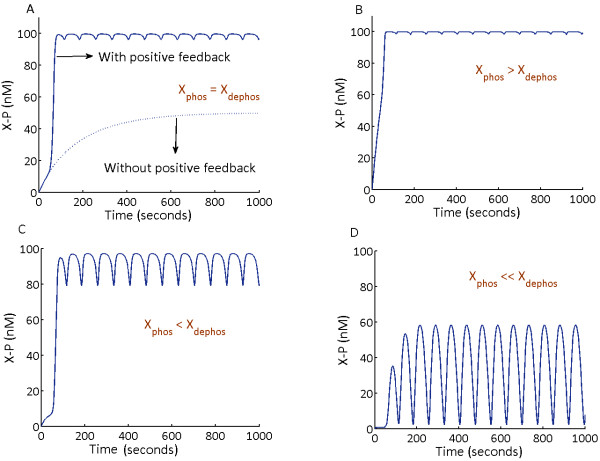
**Oscillations in an external signaling module of kinase X by the positive feedback of the MAPK cascade S2. (A)** Dynamics of X-P with and without the positive feedback loop when rate of phosphorylation of X (X_phos_ ) = rate of dephopshorylation of X (X_dephos_ ). Here X_phos_ = X_dephhos_ = 0.1. **(B)** Dynamics of phosphorylated X (X-P) when X_phos_ **>** X_dephos._ The X_phos_ = 0.5 and X _dephos_ = 0.1. **(C)** Dynamics of X-P when X_dephos._**>** X_phos._ The X_phos_ = 0.1 and X _dephos_ = 0.5. **(D)** Dynamics of X-P when X_dephos._**> >** X_phos._ The X_phos_ = 0.1 and X _dephos_ = 5. In the simulations, total concentration of X was 100 nM (arbitrary). The units of X_phos_ and X_dephhos_ is nM.sec^-1^.

We introduced the positive feedback loop from S2 to phosphorylation step of X. Simulations show that the positive feedback transfers oscillations from S2 to X where the extent of oscillations in X-P (or X) was governed by the relative rates of phosphorylation and dephopshorylation in the X module. Figure 5A shows the dynamics of X-P phosphorylation in presence and in absence of the positive feedback loops, when phosphorylation rate of X (X_phos_) is equal to dephopshorylation rate of X-P (X_dephos_). The positive feedback transferred oscillatory information from S2 to X-P together with triggering amplification in X-P amplitude. When the X_phos_ is higher than the X_dephos_ (Figure 5B) oscillatory phosphorylation of X was diluted but the amplification of X-P caused by the positive feedback remained unaffected. On the contrary when X_phos_ **<** X_dephos_ (Figure 5C), X-P exhibited oscillations with much wider differences in the maximum and minimum amplitudes of its oscillations. For significantly lower values of X_phos_ (X_phos_ < < X_dephos_), phosphorylation of X oscillated between its lowest (zero) to its maximum phosphorylation amplitude (Figure 5D). We also investigated the effect of positive feedback emerging from MAPK cascade S1 and functional in the phosphorylation step of the module X. Here too oscillations from S1 to the module X were transferred as function of relative values of X_phos_ and X_dephos_ with maximum amplitude oscillations in the X module triggered when X_phos_ **< <** X_dephos_ (data not shown)_._ This study exposes a novel cellular strategy where cells can control the effects of a positive feedback loop emerging from a MAPK cascade such as S1 or S2 and operational on different target sites. We revealed how adjustment of phosphorylation and dephosphorylation rates in the target modules would regulate the extent of oscillations in them.

Next we investigated the fate of oscillations triggered by PN-I and PN-II when nuclear cytoplasmic shuttling of the MK layer takes place. The analysis was performed to investigate the fate of oscillations triggered by PN-I and PN-II when the oscillations in the cascade output (MK**) are triggered in the cytoplasm but its nuclear translocation takes place subsequently.

### Fate of MAPK oscillations in S1 and S2 upon nuclear translocation of the MK layer followed by induction of its own nuclear phosphatase

**Figure 6:**
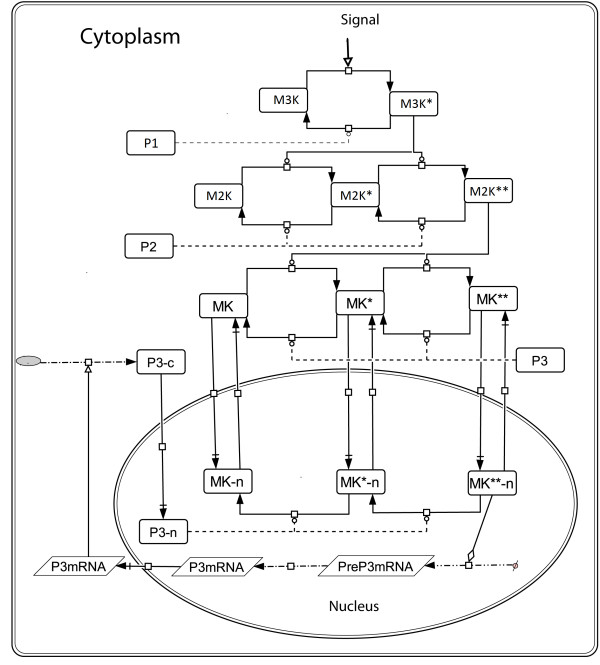
**Schematics of compartmentalized MAPK signaling and transcriptional induction of the phosphatase P3-n.** The MK, MK* and MK** comprises of the cytoplasmic fraction which translocate to the nucleus and their nuclear counterparts are MK-n, MK*-n and MK**-n. The cytoplasmic and nuclear components together constitute the total MK. MK**-n transcriptionally induces mRNA of P3-n which is shown as PreP3mRNA that finally leads to translation of P3-n in the cytoplasm (shown as P3-c). Upon nuclear translocation P3-c becomes P3-n and dephosphorylates MK**-n and MK*-n. Phosphorylation, dephosphorylation, transcription, translation, and nuclear-cytoplasmic shuffling are represented with distinct arrowheads/types and lines with blunt heads.

It was observed experimentally that upon prolonged signaling, nuclear cytoplasmic shuttling of the MK layer of the MAPK cascade takes place [10,11]. Activation of the MAPK cascade is followed by nuclear translocation of its output MK** where it induces various transcription factors including its own phosphatase. It is known that upon nuclear translocation, the doubly phosphorylated ERK** (MK** in our models) induces its nuclear phosphatase MKP-1 [10,37]. The phosphatase MKP-1 is nuclear specific; thus it dephosphorylates MK** only in the nucleus. Hence for the long duration MAPK signaling, where induction of the MAPK phosphatase MKP-1 takes place [4,37], the phosphorylated MK** is dephosphorylated in the cytoplasm by P3 and also in the nucleus by MKP-1 (P3-n in Figure 6). Here, first we investigated the sustainability of oscillations upon nuclear cytoplasmic shuttling of the MK layer components and subsequently studied the roles of P3 and induced P3-n in determining the oscillatory fate of MK** and its nuclear component MK**-n ( Figure 6). For that purpose the existing models S1 and S2 were modified to incorporate nuclear translocation of the MK layer and induction of P3-n by MK**-n (Figure 6). The modified models had 22 biochemical reactions each (S1 modified to S1n and S2 modified to S2n respectively), with the first 10 reactions in S1n and S2n being identical to S1 and S2 respectively, which are shown in Table 2. The 11 additional reactions in S1n and S2n (Shown in Table 3) captured shuttling of MK, MK* and MK** between cytoplasm and nucleus, P3-n induction steps and dephosphorylation of MK**-n and MK*-n in the nucleus by P3-n. Mechanistic and parametric details for nuclear-cytoplasmic shuttling of MK layer components and transcriptional induction of P3-n were taken from a recent study on the mammalian MAPK cascade [10]. 

### I. Oscillations in S1n

**Figure 7:**
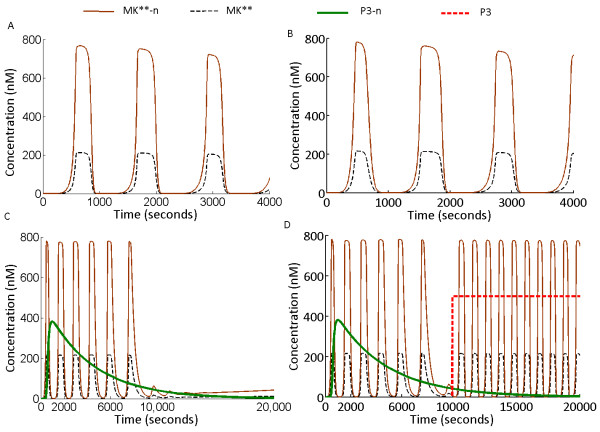
**Oscillations in S1n.** Oscillations in the MAPK cascade with underlying feedback design PN-I are not altered or abolished by its nuclear translocation and induction of the nuclear phosphatase P3-n **(A)** Oscillations in MK** and MK**-n when P3 dephosphorylated MK** in the cytoplasm and P3-n dephosphorylated MK**-n in the nucleus. **(B)** Oscillations of MK** and MK**-n when P3 was knocked out from the system, implying P3 = 0 throughout the simulations but when P3-n was allowed to be produced transcriptionally. **(C)** Oscillations in MK** and MK**-n, when P3 = 0 throughout the simulations, and P3-n production was stopped at time = 600 seconds. **(D)** P3 is knocked out initially; P3-n production was stopped at 600 seconds, which was followed by reverting the P3 concentration back to its reference value (P3 = 500 nM), at 10000 seconds.

Nuclear compartmentalization of the MK layer and transcriptional induction of P3-n didn’t affect the oscillations in S1n and it exhibited MK** oscillations with near identical frequencies as observed in S1. Nonetheless, the amplitude of cytoplasmic MK** decreased and major fraction of phosphorylated MK** resided in the nucleus (MK**-n in Figure 7A). Next we checked the roles of P3 and P3-n in deciding the oscillatory fate of MK** and MK**-n. P3 concentration was made 0 (biologically corresponds to a P3 knockout system) and the system was simulated. Figure 7B shows the results for P3 = 0, when dephosphorylation of MK**-n and MK*-n was carried out by P3-n. The simulations show that the frequency and amplitude of MK** and MK**-n were not altered when P3 is absent in the system and dephosphorylation of MK layer is carried out only in the nucleus (Compare Figure 7A and 7B). In the subsequent analysis, we stopped transcriptional induction of P3-n after 600 seconds (randomly chosen) of simulation, with P3 = 0 as an initial condition before simulation (In the model, the transcriptional induction was stopped by equating the rate of P3mRNA production to zero). We observed MK** and MK**-n oscillations for significantly long time after the transcription was stopped (Figure 7C), and only after P3-n concentration goes down a certain limit (due to its degradation), oscillations in MK** and MK**-n were abolished (Figure 7C). However oscillations could be triggered back to the system (Figure 7D) when P3 concentration was reverted from 0 back to its reference value (500 nM, Additional file 3: Table S2) after P3-n concentration goes below a value (due to its degradation) that is required to maintain sustained oscillations. The simulations thus show that MAPK cascade with architectural design such as S1n can exhibit oscillations in presence of either of the nuclear or cytoplasmic phosphatase. It could be noted that presence of both phosphatases didn’t impart any change in the frequencies and amplitudes of MK** and MK**-n.

### II. Oscillations in S2n

Simulations were carried out in S2n after incorporation of the transcriptional components (nuclear cytoplasmic shuttling and induction of P3-n) in the MAPK cascade. Similar to the model S1n, the model S2n was also built upon the existing model S2. Similar to S1n, the parameters for transcriptional processes were kept identical to the experimentally reported values [10].

**Figure 8:**
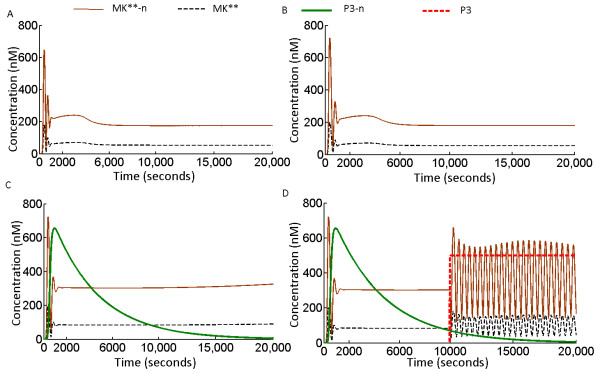
**Oscillations in S2n.** Oscillations in the MAPK cascade with underlying feedback design PN-II were terminated due to induction of its nuclear phosphatase P3-n. **(A)** Oscillations in MK** and MK**-n were abolished when P3 dephosphorylated MK** in the cytoplasm and P3-n dephosphorylated MK**-n in the nucleus. **(B)** Oscillations were not observed when P3 was knocked out (P3 = 0) and when only P3-n dephosphorylated MK**-n and MK*-n in the nucleus **(C)** For P3 = 0 throughout the simulations, and P3-n production stopped at time = 600 seconds, oscillations were not observed for any value of P3-n concentration. **(D)** When P3 was set to zero as initial condition and P3-n production was stopped at 600 seconds, followed by reverting P3 concentration back to its reference value (P3 = 500 nM) at 10000 seconds, sustained oscillations in both MK** and MK**-n were observed.

Dynamics of MK** and MK**-n phosphorylation are shown in Figure 8A. The simulations show that when MK**-n was used to induce its own phosphatase P3-n, no oscillations where observed in the system. When P3 = 0, amplitudes of MK** and MK**-n (Figure 8B), didn’t differ from the condition when P3 = 500 Nm (Figure 8A). However when only the nuclear-cytoplasmic shuttling of MK layer components was considered (without P3-n induction and keeping P3 = 500 nM), the system exhibited its characteristic oscillations (Additional file 3: Figure S1). This implies that oscillations in S2n were not abolished due to nuclear-cytoplasmic shuttling of the MK layer components, but due to the transcriptional induction of P3-n. For P3 =0 as an initial condition, followed by inhibition of P3-n at 600 seconds, the oscillations in MK** were not observed for any value of P3-n concentration (Figure 8C). Next, in a P3 knocked out system, P3-n was made zero at time = 600 seconds, followed by reverting P3 concentration back to 500 nM after time = 10000 seconds (after P3-n was significantly degraded). We found that after P3-n concentration becomes significantly low (after time = 10000 seconds) reverting P3 back to its reference value triggered sustained oscillations in both MK** and MK**-n (Figure 8D). Introduction of P3 in presence of higher concentrations of P3-n (time < = 10000) didn’t trigger oscillations in MK** and MK**-n (Additional file 3: Figure S2).

We also searched the parameter space (in the range of 0.01-100 times their reference values as given in Table 3) of model S2n for combinations of parameters that could possibly trigger sustained oscillations in S2n. The parameters were varied using Bifurcation discovery tool [23] where we searched specific combinations of parameters that could trigger oscillations in S2n in presence of both P3 and P3-n. The analysis provided a parameter set that triggered transient oscillations (data not shown), but to trigger such oscillations, values of several of the parameters were largely shifted from their experimentally observed values. Thus applying changes in those parameter values would perhaps not represent the realistic scenario anymore and we restricted ourselves from applying such changes in S2n. Our analysis thus suggests that in a MAPK cascade embedded in feedback design such as PN-II, sustained oscillations could only be triggered in absence of its nuclear phosphatase P3-n.

### PN-I and PN-II differentially shapes the MAPK cascades’ output sensitivity to small perturbations in parameter values

In signaling networks with multiple parameters, perturbation in only a few parameters pivotally decides the output fate of the systems and changes in majority of the parameters doesn’t alter the output characteristics [38]. Knowledge of the crucial and less-crucial parameter values improves the understanding on the regulatory principles and helps in finding suitable drug targets [39,40]. We subjected the kinetic parameters of S1, S2, S1n and S2n to small perturbations and the sensitivities of the outputs MK** (in S1 and S2) and MK**-n (in S1n and S2n) were calculated. Thus a model parameter ‘p’ was subjected to perturbation Δpwhere Δp= 0.001*p. Such small perturbations in the parameter values didn’t affect the sustained nature of oscillations, but revealed the relative sensitivity of the output to the perturbations.

**Figure 9:**
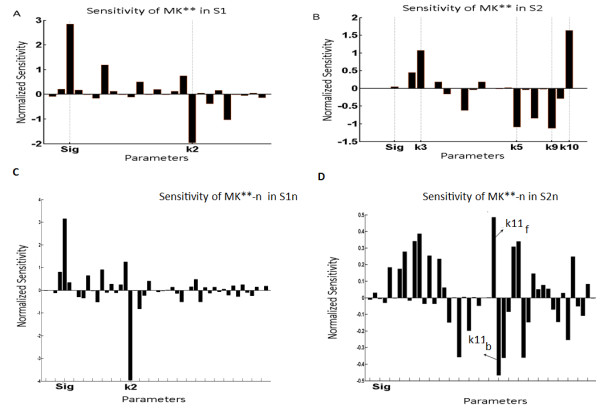
Sensitivity of MK** in S1 and S2 and MK**-n in S1n and S2n to small perturbations in the system parameters. The kinetic parameters and signal strength ‘Sig’ of both S1 and S2 were subjected to small perturbations and sensitivity of MK** (in S1 and S2) and MK**-n (in S1n and S2n) to such perturbations were calculated. **(A)** Response of MK** of the system S1 to the perturbations in kinetic parameters. **(B)** Response of MK** of the system S2 to the perturbations in kinetic parameters. **(C)** Response of MK**-n of the system S1n to the perturbations in kinetic parameters and **(D)** Response of MK**-n of the system S2n to the perturbations in kinetic parameters. The parameters perturbed are in the X axis and sensitivity of MK** (MK**-n) to such perturbations is shown in the Y axis. Only the most sensitive parameters and ‘Sig’ are shown. List of all the parameters of S1 and S2 subjected to perturbation are shown in Additional file 2: Table S1 with their respective values. List of all the parameters of S1n and S2n subjected to perturbation are listed in Table 3 of the main text.

Figure 9A and 9B shows the sensitivity of MK** to small perturbations in their model parameters. MK** in the MAPK cascade embedded in PN-I and PN-II was found to exhibit different sensitivity profiles. In the Figure 9A and 9B, only the most sensitive parameters are shown with their respective names. In S1 (Figure 9A), MK** is most sensitive to the perturbations in the strength of the incoming signal (Sig) and the dephopshorylation rate (k2) of M3K**. In S2 (Figure 9B), MK** is most sensitive to perturbations in rates of dephosphorylation in the MK layer (k9 and k10 in Figure 9B). The models S1n and S2n were also subjected to small perturbations like in S1 and S2 (Figure 9C and 9D). The sensitivity profile of MK**-n in S1n was similar to MK** in S1 with MK**-n being most sensitive to changes in signal strength and the dephosphorylation rate of M3K* (Figure 9C). MK**-n in S2n exhibited relatively higher sensitivities to the parameters involved in the shuttling of MK layer components specifically the shuttling rate of MK**-n (Figure 9D).

The differential sensitivity profile of MK** in the two models could be mechanistically understood as follows. The MAPK cascade being a ultrasensitive cascade and signal amplifier [3,25], any small changes in the input layer gets amplified as it propagates downstream and results in significantly larger changes in the output of the system. Usually negative feedback is a noise suppressor and small fluctuations in the values of signal/parameters are filtered by the negative feedback [41,42]. But as the positive feedbacks are coupled to the system as well they further amplify the effect of small changes/perturbations, and subsequently alter the phosphorylation of the MK**. Thus in S1 and S1n (Figure 9A and 9C), changes in the M3K layer due to small fluctuations in the parameter values were amplified at the M2K layer owing to the positive feedback. Thus coupling of the effect of the positive feedback together with the MAPK cascade’s inherent ability for signal amplification (due to multisite phosphorylation and multi layer organization [3,4]) resulted in maximum sensitivity of MK** to small perturbations in kinetic parameters in M3K layer. On the contrary, in S2 (Figure 9B) the incoming signal encounters the positive feedback before negative feedback. Here the changes in the M3K layer are suppressed at the M2K layer by the negative feedback but as small changes in the MK** can affect the strength of the positive feedback at the M3K layer, the output MK** exhibited maximum relative sensitivity to small changes in the MK layer itself (Figure 9B). S2n having identical architecture of feedback loops as S2 also exhibited maximum sensitivity to changes in the MK layer and the layers below MK specifically to the shuttling rate of MK** between the nucleus and cytoplasm (k11_f_ and k11_b_ in Figure 9D).

## Discussions

Computationally it was predicted more than a decade earlier that MAPK cascade can exhibit oscillations embracing one negative feedback loop from MK** to suppress M3K phosphorylation [14], much earlier than the experimental report on biochemical oscillations of the MAPK cascade [11,43]. Experiments have now shown that phosphorylation dynamics of MAPK exhibit oscillatory behavior from yeast to mammal [11,17,43]. Here we have studied the significance of differential designs of coupled positive and negative feedback loops in triggering MAPK oscillations. We have also investigated how MAPK cascades embedded in designs such as PN-I and PN-II can shape their oscillation and the effect of nuclear-cytoplasmic shuttling of the cascade components triggered by each of the design.

### Oscillations in MAPK cascade due to PN-I and PN-II designs

Although a single negative feedback is the minimal requirement for triggering MAPK oscillations, a growing number of studies indicates that oscillations in various cellular signaling systems [6,8] including the MAPK cascade [5], are triggered by coupled positive and negative feedback loops. These experimental reports led us to investigate the roles of negative and positive feedback loops operative in a three-layer MAPK cascade (Table 1). Based on literature, we found that two possible designs of coupled positive and negative feedback loops can exist in a three layer MAPK cascade (S1 and S2 in Figure 2), namely PN-I and PN-II. Our simulations show that both PN-I and PN-II can trigger oscillations in the cascade. In S1, the cascades output exhibited digital oscillations, whereas in S2 analogous oscillations were observed. These results show that the nature of the MK** output is determined by the type of the feedback loop functional in the M2K layer. From the context of information processing by a MAPK cascade, the ability to utilize two distinct designs of coupled positive and negative feedback loops would enable it to deliver unique oscillatory output while responding to input signal of similar strengths. We show that two MAPK cascades with identical concentrations of their respective kinases and phosphatases can trigger digital or analogous oscillations based on the design of coupled positive and negative feedback loop embedded in it.

Information processing systems such as the signal transduction networks are usually activated by a spectrum of signals and strength of an incoming signal may not remain constant [41,44]. Thus in the living systems a signaling pathway needs to respond to signals of various strengths and subsequently deliver the desired output. We examined whether the models S1 and S2 can deliver oscillatory output when subjected to a wide range of signal strengths. It was found that both S1 and S2 can exhibit their characteristic oscillations when subjected to a range of input signal, although the system S2 was extremely robust to increase in signal strength above a threshold. The system S1 exhibited equal amplitude oscillations whose oscillation frequencies were reciprocally dependent on the strength of the input signal (Figure 4A). However, S2 with feedback design PN-II exhibited equal amplitude and equal frequency oscillations for virtually any strength of input signal, beyond threshold signal strength (Figure 4B). As the MAPK cascade is present in almost all the living systems, it is conceivable that the cascade is subjected to signal strengths varying in orders of magnitudes. We uncovered a remarkable ability of the cascade to trigger and maintain its oscillations with unchanged amplitudes and frequencies when subjected to varying signal strengths. A recent experimental report on epithelial cells stimulated with EGF also shows that the MAPK (ERK1) cascade conserves the frequency of oscillation of MK** when subjected to perturbations [27,28]. Our analysis reveals a plausible design of coupled positive and negative feedback loops that the cascade can adopt to deliver such constant frequency oscillations. We additionally show that together with conservation of amplitude, the cascade is also capable of preserving its oscillation frequencies in response to large fluctuations in incoming signals.

Positive feedback emerging from an oscillating MAPK cascade triggers oscillations in its external target module.

Literature of intra-modular crosstalk involving MAPK pathways is abundant. In T cell receptor triggered signaling pathways, MK** is the origin of 92% of the feedback loops (both positive and negative) [19], which implies that using the positive and negative feedback loops, the MAPK cascade determines fate of multiple pathways in the large scale network. Here we showed that oscillating MAPK cascade such as S1 or S2 can use their respective positive feedback loops to trigger oscillations in any external signal transduction module. The extent of oscillation in the target module would be determined by the ratio of rates of phosphorylation and dephosphorylation in the target module. When the parametric conditions were satisfied in the target module (phosphorylation rate < dephosphorylation rate), oscillations were triggered (Figure 5C). Oscillations in the target module spanning from zero to its maximum phosphorylation amplitude were observed when phosphorylation rate was very much less than dephosphorylation rate. (Figure 5D). The ability to induce oscillations in the target modules depending on the ratio of kinetic parameters in the target module itself can be extremely useful from the cellular context. This is because a plethora of target modules, each with unique ratios of phosphorylation-dephosphorylation will differentially deliver their oscillatory outputs (Figure 5A-D).

The result also exposes a multifaceted regulatory aspect of positive feedback loops which was not specifically addressed before. Positive feedbacks hallmark characteristics is signal amplification and promoting switch like behavior to its target [25,33]. The feedbacks ability to trigger oscillations in its target (Figure 5A) reveals this novel regulatory aspect of the positive feedback.

### Fate of oscillations triggered by PN-I and PN-II upon nuclear cytoplasmic shuttling of MK layer and induction of its nuclear phosphatase

Nuclear cytoplasmic shuttling of the MAPK cascade’s MK layer components takes place and MK** induces various transcription factors including its own phosphatases [10]. The models S1 and S2 exhibited oscillations which are specific to cytoplasm but as MK layer of the cascade shuttles between the nucleus and cytoplasm, fate of the oscillations under such conditions is worth analyzing. We modified the oscillating systems where the modified systems were built with both cytoplasmic and nuclear components (S1 becomes S1n and S2 becomes S2n). The nuclear reactions comprised shuttling of MK, MK* and MK** between cytoplasm and nucleus, P3-n induction followed by dephosphorylation of MK**-n and MK*-n in the nucleus by P3-n. As the oscillations were triggered by the two different designs of feedback, PN-I and PN-II, we investigated how nuclear-cytoplasmic shuttling and transcriptional induction of P3-n affect the oscillations of S1n and S2n. Simulations show that oscillations triggered by the feedback design PN-I in S1n remains unaffected by the shuttling process and P3-n mediated dephopshorylation in the nucleus (Figure 7A-D). However oscillations in S2n were abolished when nuclear phosphatase P3-n was transcribed in the nucleus (Figure 8A-D). Hence we show for the first time that fate of oscillations in a MAPK cascade is determined by the design of coupled positive and negative feedback loops that trigger such oscillations especially when compartmentalization of the cascade components take place. The study exposed probable cellular strategies underlying generation and maintenance of robust MAPK oscillations for a longer duration, as long duration signal processing involves such nuclear cytoplasmic shuttling and activation of various transcription factors.

### The feedback designs PN- and PN-II differentially determines the MAPK cascade’s sensitivity to small perturbations in the model kinetic parameters

Local sensitivity analysis was performed to understand the responses of the outputs MK** (S1 and S2) and MK**-n (S1n and S2n) to small perturbations in their kinetic parameters (Figure 9A-D). Sensitivity analysis exposed the most sensitive parameters in the models embedded in the designs PN-I and PN-II. We found that sensitivity of MK** and MK**-n exhibits differential sensitivity profiles in S1 (S1n) and S2 (S2n), implying that the outputs sensitivity were determined by the design of the embedded feedback loops in the MAPK cascades. Sensitivity analysis results are useful for designing drugs. For example, for a system S1/S1n the most suitable strategy to suppress MK**/MK**-n will be to inhibit the strength of input stimuli (Sig) or enhance the flux of M3K* dephopshorylation. However if a drug needs to be designed for a MAPK cascade S2/S2n, MK**/MK**-n will be altered most effectively by altering the dephosphorylation flux of the MK layer (for S2) or by altering the MK layer shuffling rates (for S2n).

### Proposed experimental verification of the model propositions

The prediction made based on the simulation of the models S1, S2, S1n and S2n could be tested experimentally using different approaches. In the first approach mammalian cells such as COS-1 cells can be chosen to verify model type such S1. Experiments with COS-1 show that MK** such as ERK** gives positive feedback to M2K (MEK) phosphorylation step by inhibiting its competitive inhibitor RKIP [45]. At the same time ERK** gives negative feedback to M3K (Raf) phosphorylation by inhibiting the upstream signal that triggers Raf phosphorylation [5]. The design resembles the system design PN-I (S1) which also exhibited oscillations, as observed experimentally [5]. Hence considering COS-1 cells as experimental system one could subject them with various perturbation conditions as described in the models. For example it is predicted from the simulations that S1 can deliver oscillations with conserved amplitudes whose frequencies will vary according to the strength of incoming signal. Western blot analysis could subsequently be performed where kinetics of ERK phosphorylation for various strengths of input stimuli can be compared, which would then verify the model predictions. Further the model predicts that S1n (with design PN-I) should retain its oscillations upon nuclear-cytoplasmic shuttling and induction of phosphatase such as MKP-1 should not affect the ERK oscillations. This can be tested by subjecting the COS-1 cells to prolonged stimuli and subsequently capturing the phosphorylation kinetics of ERK**, which should exhibit oscillations, as predicted by the simulations. Presence of oscillations during the nuclear cytoplasmic compartmentalization of the ERK cascade can be experimentally tested in the same lines as explained elsewhere [11].

The system design S2 where positive and negative feedbacks are coupled as design PN-II are not reported in one single study as yet. But a recent study shows that three layer MAPK cascade can be synthetically built [44]. Such synthetic systems will be ideal for testing hypothesis. One could design the system S2 as a synthetic system. Mass spectrometry data suggest that ERK** provides positive feedback to Raf (M3K) by phosphorylating it in certain residues which enhances specificity of Raf phosphorylation by many fold [46]. Coupled to that a negative feedback from ERK to Raf can be considered in which ERK hyperphosphorylates and desensitizes Raf [47]. The overall design would resemble the system design PN-II. Here the positive feedback is in the form of enhanced Raf phosphorylation in response to the incoming signal which is followed by the negative feedback in the form of desensitization of phosphorylated Raf (M3K*) that will consequently inhibit MEK (M2K) phosphorylation. Such synthetic cascades with positive and negative feedback resembling design PN-II could be subjected to signals of variable strengths and the oscillatory amplitudes of the cascade output can be captured in the form of western blots. The simulations proposed that the system S2 subjected to a very wide range of input signal should exhibit oscillations with conserved amplitude and frequencies which could be verified building the synthetic MAPK cascade.

### Biological significance of MAPK oscillations and proposed implications of this study

Exact biological message encoded in the oscillatory waves of the MAPK cascade is not yet understood well, though it is argued that the oscillatory MK** fulfils some requirement for triggering transcription of certain cyclic genes [48]. The current archetype states that, signaling system in general encodes messages either in amplitude or in frequency (or may be in both) of the oscillatory signals, for triggering transcription of a plethora of genes [11,12,24,48]. Here, through our study we demonstrated various ways in which unique oscillatory message could be transmitted by the MAPK cascade embedded in coupled positive and negative feedback loops to its nuclear targets.

The feedback design PN-I can trigger oscillations of equal amplitudes but of variable frequencies. This type of cascade could be utilized by the cell for activating a subset of cyclic target genes, all of which require identical amplitude of MK** as their activation threshold but the interval of expression of each target gene is determined by the frequency of oscillations. The feedback design PN-II can be utilized to deliver oscillations with near identical frequency and amplitude in response to widely varying signal strengths. This type of feedback design would be suitable for a MAPK cascade involved in robustly inducing specific sets of genes whose expressions are critically dependent on the amplitude and/or frequencies of the MK**.

We demonstrated how oscillations could be maintained during a long duration signaling when signal processing involves nuclear-cytoplasmic shuttling of the MK layer of the cascade, followed by transcriptionally inducing the phosphatases that interact with the cascade itself. We showed that it is not always possible to maintain oscillations in the face of obvious biological perturbations, such as interaction with the transcriptionally induced phosphatases and thus the cascade has to adopt certain feedback designs (such as PN-I) to endure such perturbations to exhibit prolonged oscillations.

## Conclusion

The MAPK cascade can utilize architecturally distinct organizations of coupled positive and negative feedback loops to trigger its oscillations. We uncovered that the signaling pathways such as the MAPK pathway can uniquely process wide range of signals by utilizing its feedback loops. It is intriguing how adoption of specific design (PN-II) of coupled feedback loops can trigger oscillations with extremely robust frequency and amplitude, specifically when such robustness in the oscillations are desired in an environment where the external signal strength fluctuates in several orders of magnitudes. Subsequently we show the trade off associated with such feedback designs (PN-II) during the nuclear cytoplasmic compartmentalization of the cascade, where oscillations triggered by PN-II couldn’t sustain such compartmentalization effect. However oscillations triggered by PN-I were robustly maintained during the compartmentalization of the MAPK cascade components. Thus it can be argued based on our analysis that MAPK cascade embedded in PN-II can be used by specific cell types to exhibit short duration oscillations in response to extremely noisy signal, where frequency and amplitude needs to be robustly maintained. The oscillations triggered by PN-II will be of short duration as longer duration in signaling implies nuclear compartmentalization of the MAPK cascade, which leads to attenuation of PN-II triggered oscillations. On the contrary the design PN-I can trigger long duration oscillations (involving nuclear cytoplasmic compartmentalization), when the cascade embedded in such design is exposed to a relatively less noisy input signal.

We additionally found a completely unexpected regulatory behavior of the positive feedback component of a coupled positive and negative feedback loop used for triggering MAPK oscillations. We show that positive feedback emerging from an oscillating MAPK cascade can generate a spectrum of unique oscillatory information to various external target modules. The amplitude of oscillations thus triggered would depend on the ratio of phosphorylation and dephosphorylation in each of the target modules, which means, each target can attain differential oscillatory fates by adjusting such ratios.

## Availability of supporting data

The supporting data are provided as additional files with the manuscript. Additional files include Additional file 3: Figure S1, Additional file 3: Figure S2, two Additional tables and five SBML models.

## Competing interests

The authors declare that they have no competing interests.

## Authors' contributions

US and IG initiated the study. US did the model building, performed the simulations and did the analysis. US and IG organized the results and wrote the manuscript. All authors read and approved the final manuscript.

## Supplementary Material

Additional file 1 **SBML model files.Model S1.** SBML model of the system S1 as described in the main text. The model file is provided as ‘S1.xml’. **Model S2.** SBML model of the system S2 as described in the main text. The model file is provided as ‘S2.xml’. Positive feedback from S2 triggers oscillations in external signal transduction module. SBML model of the system S2 where MK** of S2 provides positive feedback to phosphorylation step of a kinase X as described in main text and Figure 5. The model file is provided as ‘S2_external_crosstalk.xml’. **Model S1n.** SBML model of the system S1n as described in the main text. The model file is provided as ‘S1n.xml’. **Model S2n.** SBML model of the system S2n as described in the main text. The model file is provided as ‘S2n.xml’. Click here for file

Additional file 2 **Table S1.** Flux of signal flow and the values of kinetic parameters used for simulation of S1, S2, S1n and S2n. In the Table, Ki, i= 1–10 are the Km values of the reactions and ki, i= 2–10 are the kcat values of the reactions. The numerical value of ‘i’ corresponding to Ki and ki represents the reaction number. KI are the kinetic parameters associated with negative feedback. Ka and A are the kinetic constants associated with the positive feedback. The hill coefficient used in the equations 1, 3 and 4 are shown as n1, n3 and n4 respectively. **Table S2.** Initial concentrations of the kinases and phosphatases used in the models S1, S2, S1n and S2n. Click here for file

Additional file 3 **Figure S1.** Oscillation in S2n when transcriptional induction of P3-n was stopped. P3 was present in the system (P3 = 500 nM) but P3-n induction was not considered during the course of simulation. Nuclear-cytoplasmic shuttling of the MK, MK* and MK** was considered. **Figure S2.** Oscillation couldn’t be triggered in S2n before P3-n concentration goes down significantly. Initially, P3 = 0 and P3-n production was stopped at time = 600 seconds. P3 concentration was reverted back to 500 nM at time = 2000 seconds. Oscillations were not triggered. Click here for file
