# Supplementary material for: Oscillations in MAPK cascade triggered by two distinct designs of coupled positive and negative feedback loops
Source: BMC Res Notes. 2012 Jun 13;5:287. doi: 10.1186/1756-0500-5-287 (PMC3532088; doi:10.1186/1756-0500-5-287)
Supplement: Additional file 2 — Table S1. Flux of signal flow and the values of kinetic parameters used for simulation of S1, S2, S1n and S2n. In the Table, Ki, i= 1–10 are the Km values of the reactions and ki, i= 2–10 are the kcat values of the reactions. The numerical value of ‘i’ corresponding to Ki and ki represents the reaction number. KI are the kinetic parameters associated with negative feedback. Ka and A are the kinetic constants associated with the positive feedback. The hill coefficient used in the equations 1, 3 and 4 are shown as n1, n3 and n4 respectively. Table S2. Initial concentrations of the kinases and phosphatases used in the models S1, S2, S1n and S2n. [file 1756-0500-5-287-S2.doc]

**Additional Tables**

**1] Additional table 1:**

Flux of signal flow and the values of kinetic parameters used for simulation of S1, S2, S1n and S2n. In the Table, Ki, i= 1-10 are the Km values of the reactions and ki, i= 2-10 are the kcat values of the reactions. The numerical value of ‘i’ corresponding to Ki and ki represents the reaction number. KI are the kinetic parameters associated with negative feedback. Ka and A are the kinetic constants associated with the positive feedback. The hill coefficient used in the equations 1, 3 and 4 are shown as n1, n3 and n4 respectively.

| **Reactions** | **Parameter values in S1 and S1n** | **Parameter values in S2 and S2n** |
| --- | --- | --- |
| 1]  M3K 🡪 M3K* | Sig = 2.5 nM/sec  K1 = 20 nmol/ml  KI = 9 nmol/ml  n1 = 1 | Sig = 6 nM/sec  K1 = 15 nmol/ml  A = 100  Ka = 500 nmol/ml  n1= 1 |
| 2]  M3K* 🡪 M3K | K2 = 200 nmol/ml  k2 = 0.025 sec^-1^ | K2 = 100 nmol/ml  k2=0.1 sec^-1^ |
| 3]  M2K 🡪 M2K* | K3 = 20 nmol/ml  k3 = 0.1 sec^-1^  A = 10  Ka = 500 nmol/ml  n3 = 1 | K3 = 20 nmol/ml  k3=0.1 sec^-1^  KI = 9 nmol/ml  n3 = 1 |
| 4]  M2K* 🡪 M2K** | K4 = 20 nmol/ml  k4 = 0.1 sec^-1^  A = 10  Ka = 500 nmol/ml  n4 = 1 | K4 = 20 nmol/ml  k4=0.1 sec^-1^  KI = 9 nmol/ml  n4 = 1 |
| 5]  M2K** 🡪 M2K* | K5 = 200 nmol/ml  k5 = 0.1 sec^-1^ | K5 = 20 nmol/ml  k5 = 0.02 sec^-1^ |
| 6]  M2K* 🡪 M2K | K6 = 200 nmol/ml  k6 = 0.1 sec^-1^ | K6 = 20 nmol/ml  k6 = 0.02 sec^-1^ |
| 7]  MK 🡪 MK* | K7 = 20 nmol/ml  k7 = 0.1 sec^-1^ | K7 = 20 nmol/ml  k7 = 0.1 sec^-1^ |
| 8]  MK*🡪 MK** | K8 = 20 nmol/ml  k8 = 0.1 sec^-1^ | K8 = 20 nmol/ml  k8 = 0.1 sec^-1^ |
| 9]  MK** 🡪 MK* | K9 = 200 nmol/ml  k9 = 0.1 sec^-1^ | K9 = 20 nmol/ml  k9 = 0.02 sec^-1^ |
| 10]  MK* 🡪 MK | K10 = 200 nmol/ml  k10 = 0.1 sec^-1^ | K10 = 20 nmol/ml  k10 = 0.02 sec^-1^ |

**2] Additional table 2: Concentrations of the kinases and phosphatases studied in S1, S2, S1n and S2n**.

| **Species** | **Concentration (nM)** |
| --- | --- |
| M3K | 1000 |
| M2K | 4000 |
| MK | 1000 |
| P1 | 100 |
| P2 | 500 |
| P3 | 500 |
