# Supplementary figures and images for: Oscillations in MAPK cascade triggered by two distinct designs of coupled positive and negative feedback loops
Source: BMC Res Notes. 2012 Jun 13;5:287. doi: 10.1186/1756-0500-5-287 (PMC3532088; doi:10.1186/1756-0500-5-287)

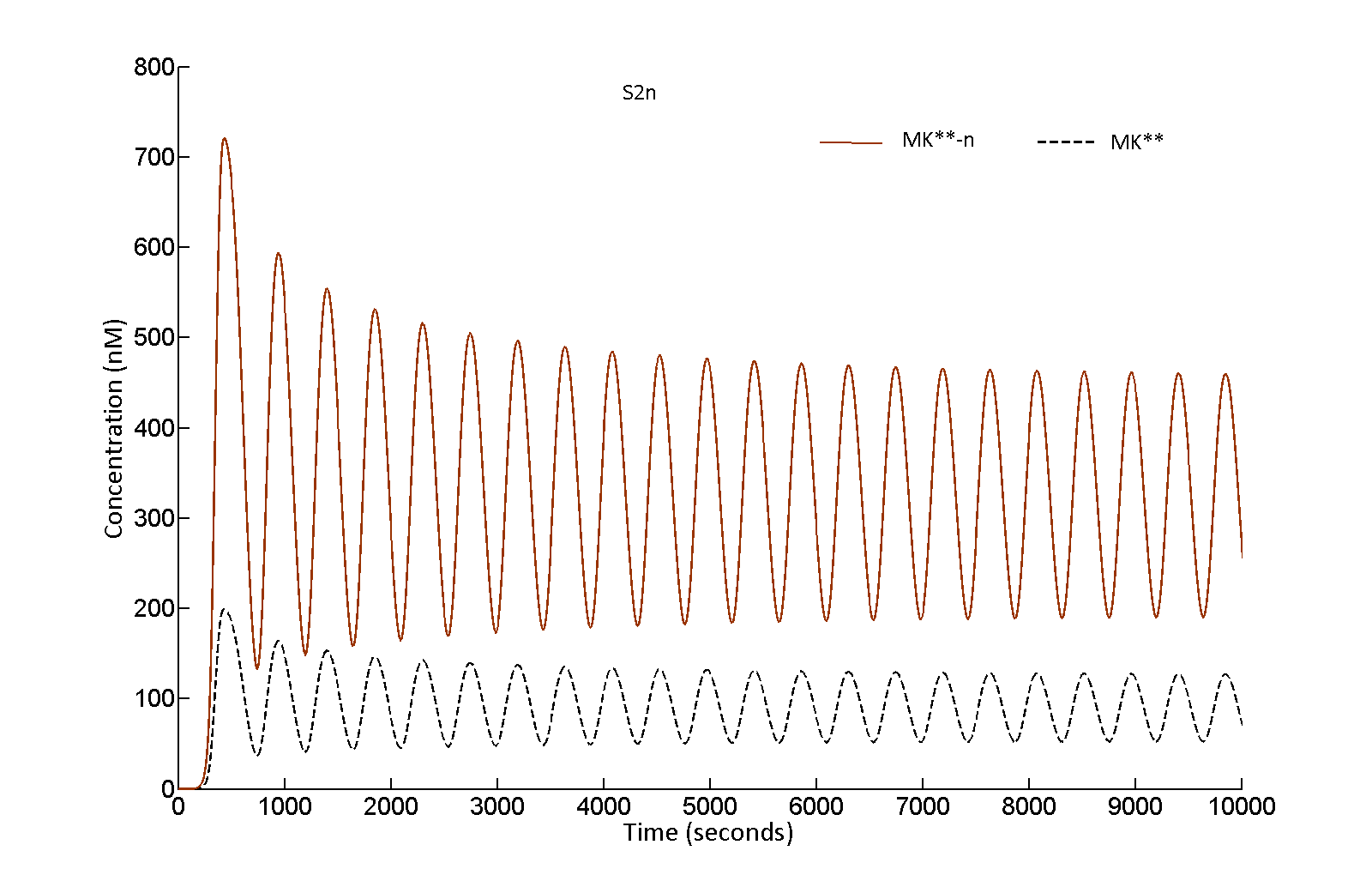

Supplement: Additional file 3 — Figure S1. Oscillation in S2n when transcriptional induction of P3-n was stopped. P3 was present in the system (P3 = 500 nM) but P3-n induction was not considered during the course of simulation. Nuclear-cytoplasmic shuttling of the MK, MK* and MK** was considered. Figure S2. Oscillation couldn’t be triggered in S2n before P3-n concentration goes down significantly. Initially, P3 = 0 and P3-n production was stopped at time = 600 seconds. P3 concentration was reverted back to 500 nM at time = 2000 seconds. Oscillations were not triggered. [file 1756-0500-5-287-S3.zip › 12889_5331704526497660_MOESM7_ESM.tiff]

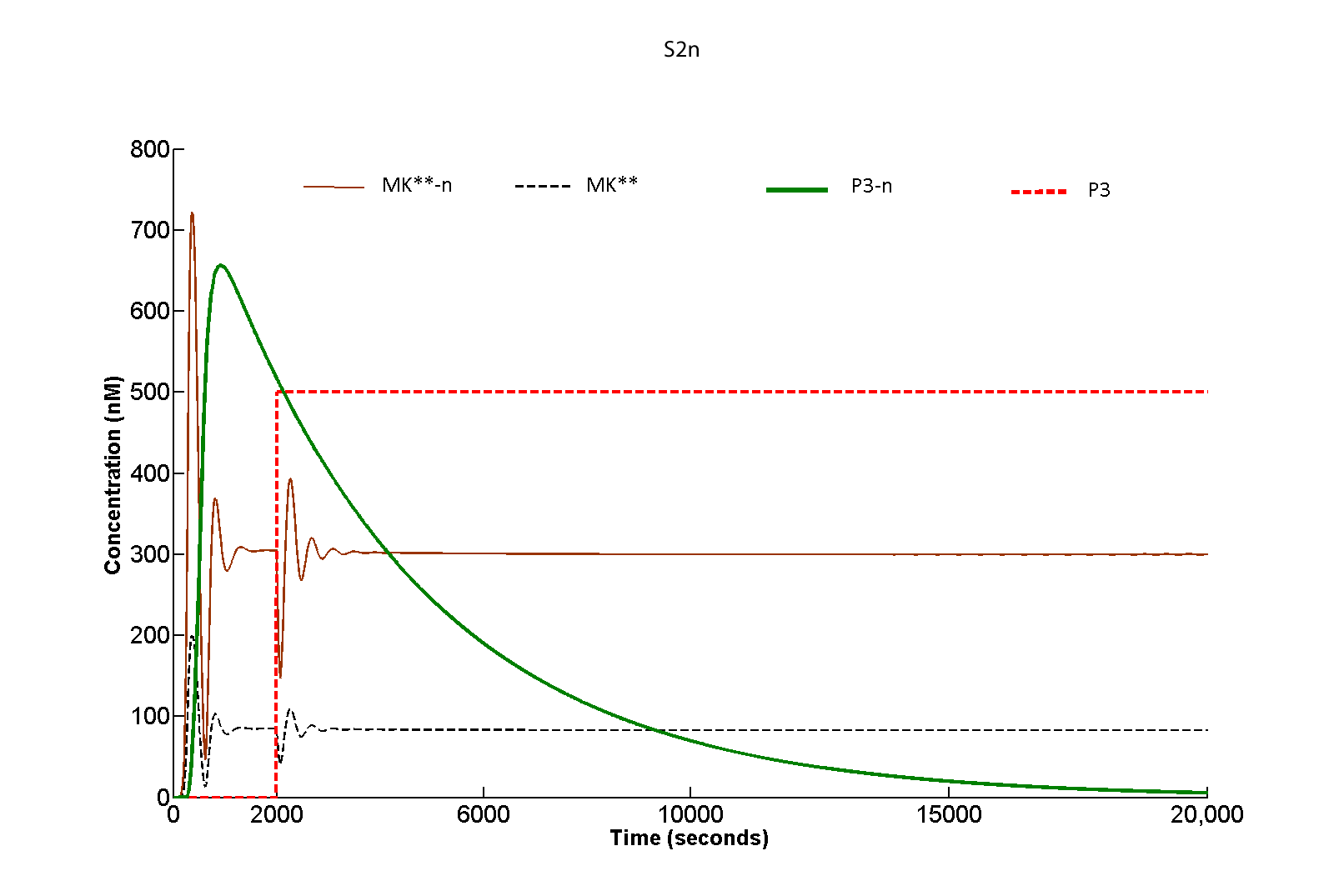

Supplement: Additional file 3 — Figure S1. Oscillation in S2n when transcriptional induction of P3-n was stopped. P3 was present in the system (P3 = 500 nM) but P3-n induction was not considered during the course of simulation. Nuclear-cytoplasmic shuttling of the MK, MK* and MK** was considered. Figure S2. Oscillation couldn’t be triggered in S2n before P3-n concentration goes down significantly. Initially, P3 = 0 and P3-n production was stopped at time = 600 seconds. P3 concentration was reverted back to 500 nM at time = 2000 seconds. Oscillations were not triggered. [file 1756-0500-5-287-S3.zip › 12889_5331704526497660_MOESM8_ESM.tiff]
